# Caucasian Infants’ Attentional Orienting to Own- and Other-Race Faces

**DOI:** 10.3390/brainsci10010053

**Published:** 2020-01-17

**Authors:** Jonathan E. Prunty, Kelsey C. Jackson, Jolie. R. Keemink, David J. Kelly

**Affiliations:** 1 School of Psychology, Keynes College, University of Kent, Canterbury, Kent CT2 7NP, UK; j.e.prunty@kent.ac.uk (J.E.P.); jrk26@kent.ac.uk (J.R.K.); 2 Seattle Children’s Innovative Technologies Lab, Seattle Children’s Hospital, Seattle, WA 98105, USA; kelseyjacks@gmail.com

**Keywords:** eye movements, development, vision, face processing, race

## Abstract

Infants show preferential attention toward faces and detect faces embedded within complex naturalistic scenes. Newborn infants are insensitive to race, but rapidly develop differential processing of own- and other-race faces. In the present study, we investigated the development of attentional orienting toward own- and other-race faces embedded within naturalistic scenes. Infants aged six-, nine- and twelve-months did not show differences in the speed of orienting to own- and other race faces, but other-race faces held infants’ visual attention for longer. We also found a clear developmental progression in attentional capture and holding, with older infants orienting to faces faster and fixating them for longer. Results are interpreted within the context of the two-process model of face processing.

## 1. Introduction

Faces represent a unique and important class of social stimuli. Humans possess a dedicated neural system for processing faces [1] and adults rapidly orient toward faces within their environment [2]. Human infants show a preference for faces over other objects [3,4] and will orient towards face-like stimuli just after [5,6] and even prior to birth [7], but see [8]. The dominant theory accounts for this innate predisposition to orient to faces through a two-process model [9]. Faces located in the visual periphery are rapidly foveated, or ‘detected’, via a subcortical system termed *Conspec*, which is considered to be functional across the lifespan. This system adaptively biases visual input toward faces, ensuring the specialisation of the cortical system, termed *Conlern*, which is responsible for ‘higher order’ aspects of face processing, such as attention, preference and recognition. 

Despite the importance of the face orienting system in development, little is known about which factors might influence it. To date, just a small number of studies have actively explored the extent to which infants preferentially orient to faces when presented alongside competing stimuli. The face orienting bias can be assessed by measuring how quickly infants fixate a face embedded within a scene (attention *capture*), and whether the infant’s *first* fixation following stimulus onset prioritises the face over other potential locations (face *detection*). In addition to orienting, attentional biases for faces can also be measured by how much time infants spend looking at the face relative to other non-face stimuli (attention *holding*). Studies that have explored these behaviours have consistently found that infants show greater attentional holding for faces, but findings for face orienting are mixed see [10,11]. 

Six-month-old infants reliably *detect* faces within circular arrays containing various objects as distractors, regardless of their orientation [12,13,14]. However, using grayscale images, non-human primate faces or varying the number of distractors in a display has been shown to impact performance [15,16]. Studies that have used more realistic scenes rather than circular arrays or grids have found increasing sensitivity to faces across the first year [17,18]. Collectively, these studies suggest that developing attentional abilities [18], oculomotor control and sociodemographic factors might also impact an infant’s ability to orient to a face [17]. A recent study reported that infants aged three- to twelve-months show consistent *detection* (as measured via first-look orienting) of faces in complex real-world scenes. This suggests that infant face orienting might be improved within naturalistic scenes compared to more artificial presentations [19], highlighting the role of context and visual experience in guiding fixations toward salient objects such as faces.

Although a picture of the development of infant biases for face orienting and attention holding is emerging, no study to date has actively investigated whether these abilities differ according to stimulus race. This is surprising given infant preferences and recognition abilities for own- and other-race stimuli have been extensively studied see [20] for a recent review. For instance, we know that while newborn infants are insensitive to race [21,22], by three months of age they show spontaneous preferences for own-race faces [21,23,24], and their ability to discriminate between other-race faces declines across the first year [2,3,4,6,9,10,12,13,14,15,16,18,19,23,24,25,26,27,28,29,30,31,32,33,34,35]. 

Moreover, from six months of age, infants begin to categorise faces according to race [25,36], and by nine months infants show a broad categorisation of different classes of other-race faces within habituation-response paradigms. For example, Caucasian infants viewed African and Asian faces according to a single ‘other’ category [36]. This conceptual shift mirrors the decline in the ability to recognise other-race faces [33,34], and coincides with the emergence of an attentional preference for other-race faces [29,37,38]. Similar to the finding that adults can more rapidly locate a lone other-race face amongst own-race distractors [39], nine-month-olds also show a visual preference for an eight-item display containing seven own-race faces and one other-race face versus an eight-item display containing seven other-race faces and one own-race face [31]. Taken together, this literature suggests that by nine months, infants may exhibit an attentional bias to, as well as rapidly categorise, other-race faces. Yet asymmetries in attention *holding* for own- and other-race faces may not necessarily generalise to face orienting given that this process is mediated by a ‘broadly-tuned’ subcortical pathway (*Conspec*) sensitive to low spatial frequency information, such that preferential orienting occurs even for face-like configural stimuli [9,10]. 

In the present study, we investigated this possibility by examining the development of attentional bias toward own- and other-race faces embedded within naturalistic scenes. Given that we are primarily interested in the capacity of infants to orient to faces, rather than to people per se, each stimulus contains a ‘disembodied’ face that appears in an ‘unexpected’ location. This approach converges with other studies exploring face detection in adult observers e.g., [26,30], and allows us to rule out the possibility that infants’ gaze orienting was biased by the presence of a body, which can act as a contextual cue, by guiding the visual system to the location of a face e.g., [27]. Specifically, we will assess if infants six, nine and twelve months demonstrate differences in attention *capture* and attention *holding* (see Table 1) for own- and other-race faces. We define attention *capture* (i.e., orienting) as the time taken to orient to a face following stimulus presentation onset and we define attention *holding* as the percentage of time spent looking at the face once it has been fixated. Additionally, following Kelly et al. [19], we will also investigate if there are any racial asymmetries in face *detection*, which we define as the number of instances (i.e., trials) in which the infant’s first saccade is directed to the face following stimulus onset. 

## 2. Methods

### 2.1. Participants

Participants were contacted via the Kent Child Development Unit database following initial recruitment at local mother and baby groups. Infants were deemed eligible to participate if they were within a ±14 days age range of a target age at the time of testing. Infants with any known visual impairments were considered ineligible for the study and not invited for testing. The participants’ caregivers were provided with an information sheet prior to testing and additionally given the opportunity to verbally ask questions before signing a consent form. Participants and caregivers were compensated with age-appropriate gifts. The study had full ethical approval from the School of Psychology’s Ethics Committee. 

A total of 172 Caucasian infants were included in the analyses with a further 12 infants excluded for failing to produce useable data (6 months, *n* = 3; 9 months, *n* = 7; 12 months, *n* = 2). Infants were omitted from the final sample for failing to complete a minimum of 8 trials (*n* = 9) or providing unanalysable data (*n* = 3) as a consequence of extreme movement. This final sample comprised infants from three distinct age groups: 6 months (*n* = 50), 9 months (*n* = 73) and 12 months (*n* = 49; See Table 2). The ethnic population of Kent is not diverse, with the latest available census data showing that 92.7% of the population categorised themselves as white and just 1.3% of the population categorised themselves as black (Office for National Statistics, 2011). 

### 2.2. Stimuli

**Figure 1:**
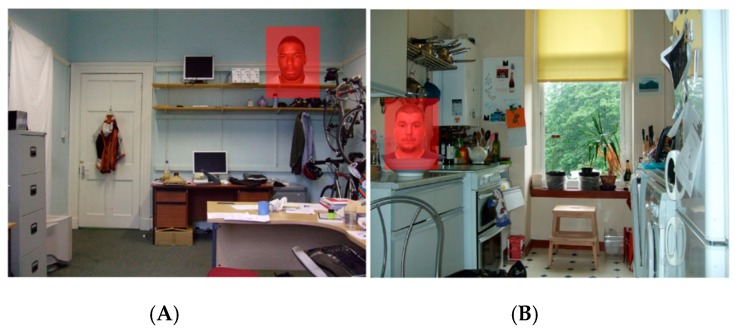
Examples of black (**A**) and white (**B**) face stimuli with AOI regions used for analyses overlaid.

Stimuli were constructed by embedding black faces (*n* = 8; 4 × male, 4 × female) and white faces (*n* = 8; 4 × male, 4 × female), taken from the Minear and Park [40] face database, within photographs of complex indoor scenes that had been used in a previous study [27]. Faces taken from the Minear and Park database held a neutral expression and were cropped to remove all background details, scaled to a size of 250 × 150 pixels and were embedded within a scene measuring 1024 × 768 pixels. Faces were placed in random locations to ensure that infants’ could not predict their location, although 4 faces appeared on the right side of the screen and 4 faces appeared on the left side of the screen in each condition. Faces subtended a size of 9.0° × 5.4° and the screen subtended a size of 37.2° × 28.9°. Example stimuli are displayed in Figure 1. The vector distance from screen centre to face centre varied slightly between individual images, but were equivalent across black and white face stimulus sets (black faces: *M* = 13.92°, *SD* = 2.27; white faces: *M* = 13.88°, *SD* = 1.89; *t*(14) = 0.036, *p* = 0.972; See Table S1). 

### 2.3. Materials

Eye movements were recorded with an Eyelink 1000+ (SR Research, Ontario) at a sampling rate of 500 Hz operated in Head Reference Mode using a 25 mm lens attachment. Infants aged 12 months were tested using the 890 nm illuminator, while all other age groups were tested using the 940 nm illuminator. Under optimal conditions, when operating in Remote Mode the Eyelink has accuracy of 0.5°, a tracking range of 32° (horizontal) × 25° (vertical) and is tolerant to head movements of 22 × 18 × 20 cm. In order to minimise head movements, infants were securely fastened in an age-appropriate car seat that was safely attached to a chair. 

The stimuli were presented using Experiment Builder (SR Research, Ontario, CA) and the raw eye movement data were extracted using Data Viewer (SR Research). Fixations and saccades were subsequently parsed in Matlab (The Mathworks, MA, USA) using custom written code. All subsequent data processing was completed using further custom written Matlab code. 

### 2.4. Fixation and Saccade Parsing

A custom-written velocity-based algorithm was used to identify saccades. Data was initially smoothed by applying a four-sample rolling window that returned a median average. Angular speed was computed based on four samples. Velocity values greater than 1000°/sec were judged to be impossible and were removed from analysis. We set a velocity threshold of 40°/sec, with samples falling below this value identified as potential fixation samples. Time and distance between two potential fixations were calculated. If inter-fixation values were <20 ms and <0.03° then fixations were merged. All fixations <100 ms were removed. Following Holmqvist, Nystrom and Mulvey [32], precision values were calculated as the root mean square (RMS) of sample-to-sample distances within computed fixations. Precision was calculated separately for each age group and results were as follows: 6 months = 0.62° (*SD* = 0.09°), 9 months = 0.57° (*SD* = 0.07°) and 12 months = 0.58° (*SD* = 0.09°). In order to assess looking to faces, an area of interest (AOI) measuring 250 × 150 pixels (9.0° × 5.4°) was placed over each face (See Figure 1). All fixations located within these spatial regions were considered to represent face looking. 

### 2.5. Procedure

The caregivers of the participants were greeted and taken to a waiting room. After signing the consent form, caregivers and infant participants were escorted to the research laboratory. Infants were placed in an age-appropriate padded seat in front of a computer monitor positioned at a distance of 60 cm. Testing was conducted in low light conditions. In order to operate the Eyelink in Remote Mode, a small target sticker was placed centrally on the infant’s forehead. The target serves as an external reference point to the tracked eye. The infant’s right eye was tracked throughout testing. The infant’s view to their surroundings, caregiver/s and experimenters was obstructed by an occluding screen in order to minimize distractions. A 5-point calibration procedure using custom-made attention-grabbing audio-visual targets was conducted initially and repeated as many times as required. No infant failed to calibrate. Following successful calibration (calibration-validation error < 1°), the task was immediately initiated. The sixteen test images were presented sequentially for 5 s each in a fully randomised order. An attention grabber appeared in the centre of the screen between each stimulus presentation that centred the infant’s gaze for the beginning of each trial. The trial was initiated only when the infant’s fixation fell within 1° of the target, thus ensuring that the infant was fixating the screen centre at the start of each trial. Including the calibration procedure, the total testing time for each infant was no longer than 3 min.

## 3. Results

### 3.1. Attention Capture

**Figure 2:**
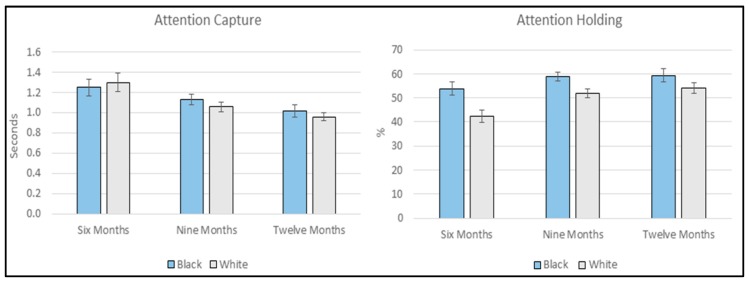
Face Fixation Onset Time (*Attention Capture*) and Post-First-Face Fixation Dwell Time (*Attention Holding*) for own- and other-race faces divided by age. Error bars are standard error of the mean.

To investigate differences in attention *capture* to own- and other-race faces, a 3 (Age: 6-, 9-, or 12-months) × 2 (FaceRace: black or white) mixed ANOVA was conducted. One six-month-old infant failed to look at any white faces and was accordingly omitted from the analysis. The ANOVA yielded a main effect of Age (*F*(2, 168) = 7.758, *p* < 0.001, ŋ_p_^2^ = 0.085), but neither the main effect of FaceRace (*F*(1, 168) = 0.361, *p* = 0.549, ŋ_p_^2^ = 0.002) nor the interaction (*F*(2, 168) = 0.718, *p* = 0.489, ŋ_p_^2^ = 0.008) reached significance. Post-hoc Bonferroni corrected comparisons found that 6-month-old infants were slower to orient to faces relative to both 9- (*p* = 0.009) and 12-month-old infants (*p* < 0.001). No other age comparisons reached significance (see Figure 2 and Table S2). 

### 3.2. Attention Holding

To investigate differences in attention *holding* for own- and other-race faces once they had been initially fixated, a 3 (Age: 6-, 9-, or 12-months) × 2 (FaceRace: black or white) mixed ANOVA was conducted. Attention holding was necessarily converted to a percentage value to prevent this analyses being confounded by differences in initial looking time. The ANOVA yielded a main effect of Age (*F*(2, 169) = 5.168, *p* = 0.007, ŋ_p_^2^ = 0.058) and a main effect of FaceRace (*F*(1, 169) = 34.862, *p* < 0.001, ŋ_p_^2^ = 0.171) but the interaction failed to reach significance (*F*(2, 169) = 1.909, *p* = 0.151, ŋ_p_^2^ = 0.022). Post-hoc Bonferroni corrected comparisons found that 6-month-old infants differed significantly from both 9- (*p* = 0.023) and 12-month-old infants (*p* = 0.011). No other age comparisons reached significance. Inspection of means (See Figure 2 and Table S2) showed that infants looked longer toward black faces relative to white faces. 

### 3.3. Face Detection 

**Figure 3:**
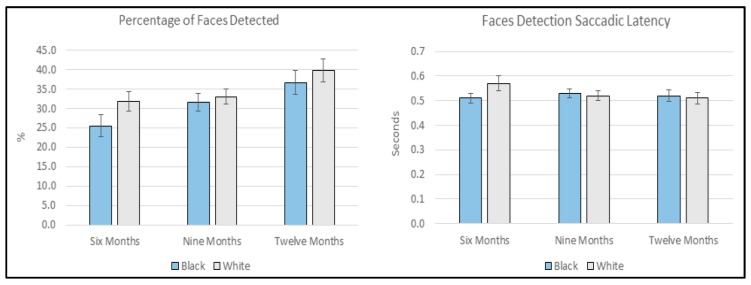
The percentage of total faces (8 per race condition) that were detected and saccadic latency for face detection (seconds) for own- and other-race faces divided by age. Error bars are standard error of the mean.

In order to explore the percentage of faces that were *detected*, a 3 (Age: 6-, 9-, or 12-months) × 2 (FaceRace: black or white) mixed ANOVA was conducted. The ANOVA yielded a main effect of Age (*F*(2, 169) = 4.637, *p* = 0.011, ŋ_p_^2^ = 0.052) and a main effect of FaceRace (*F*(1, 169) = 4.585, *p* = 0.034, ŋ_p_^2^ = 0.026) but the interaction failed to reach significance (*F*(2, 169) = 0.699, *p* = 0.499, ŋ_p_^2^ = 0.008). Post-hoc Bonferroni corrected comparisons found that 6-month-old infants detected significantly fewer faces relative to 12-month-old infants (*p* = 0.009) and inspection of means (See Figure 3 and Table S3) showed white faces (*M* = 34.50%, *SD* = 18.37) were more likely to be detected relative to black faces (*M* = 31.25%, *SD* = 20.50). No other age comparisons reached significance. 

Following Gluckman and Johnson [14] and Kelly et al. [19], the percentage of first-looks to face AOIs were computed to assess whether faces had been prioritised over other information contained within each scene. Unlike in the adult literature (e.g., [6]) where the contributions of bottom-up and top-down factors have been studied extensively, it is currently unclear how these factors influence infant looking, thus no location on the screen was considered more or less likely than any other location to attract the initial fixation. Accordingly, we contrasted the percentage of first-looks to face AOIs (% of total) against chance (5.58%—the total pixel space occupied by the face AOI), assuming an equal probability of looking toward any location on the stimulus. Face detection was significantly above chance for six- (Black: *t*(49) = 7.03, *p* < 0.001; White: *t*(49) = 10.35, *p* < 0.001), nine- (Black: *t*(72) = 11.33, *p* < 0.001; White: *t*(72) = 14.50, *p* < 0.001), and twelve-month (Black: *t*(48) = 10.12, *p* < 0.001; White: *t*(48) = 12.25, *p* < 0.001) age groups. 

### 3.4. Face Detection Saccadic Latency 

We then further investigated face *detection* by conducting a 3 (Age: 6-, 9-, or 12-months) × 2 (FaceRace: black or white) mixed ANOVA on the latency of face-targeting initial saccades. Neither the main effect of Age (*F*(2, 146) = 0.578, *p* = 0.562, ŋ_p_^2^ = 0.008), nor FaceRace (*F*(1, 146) = 1.684, *p* = 0.196, ŋ_p_^2^ = 0.011), nor the interaction (*F*(2, 146) = 1.633, *p* = 0.199, ŋ_p_^2^ = 0.022) reached significance. Twenty-three infants (6M *n* = 11, 9 M *n* = 10, 12 M *n* = 2) were excluded from this analysis as they did not *detect* a minimum of one face from each race (See Figure 3 and Table S3).

### 3.5. Image Analysis: Face Location 

In order to determine if face orienting and detection differed according to the location of the face within each stimulus and critically, that the differences reported for *detection* above were not driven by face location differences between stimulus sets, the distance (in degrees of visual angle) of the face from the image centre was computed and compared to the total number of trials where faces were *detected* (i.e., the first saccade of that trial targeted the face) for each stimulus image (*N* = 16) and *captured* (i.e., fixated during the trial time period). ANCOVAs were conducted with Age and FaceRace as categorical variables and Distance as a continuous variable. The *face detection* ANCOVA yielded a main effect of Distance (*F*(1, 38) = 21.481, *p* < 0.001, ŋ_p_^2^ = 0.361), with faces closer to the screen centre being detected more frequently than faces situated more distantly from the screen centre, but neither the main effects of Age (*F*(2, 38) = 21.481, *p* = 0.576, ŋ_p_^2^ = 0.029) or FaceRace (*F*(1, 38) = 1.409, *p* = 0.243, ŋ_p_^2^ = 0.036) nor any interaction reached significance suggesting that the race-related differences in detection rates reported in the main analyses above were not driven by differences in face location across FaceRace conditions. The *face capture* ANCOVA yielded no main effects, suggesting face location had no impact on this behaviour. A final ANCOVA contrasted the speed of face orienting (*attentional capture*) in relation to face distance, but it also failed to produce any significant effects, suggesting that the time it took infants to direct an initial saccade to a face was not related to face location. Full details of face distance alongside detection number, capture number and orienting speed for each image can be found in Table S3 in the supplementary information.

### 3.6. Image Analysis: Visual Saliency 

As a final check, we assessed whether any low-level perceptual differences in visual saliency between stimulus sets could account for the findings reported above. Each individual was analysed for its visual content using the Visual Saliency Toolbox [41], which assesses the low-level visual properties (e.g., brightness, contrast etc.) of images and produces a corresponding map highlighting the most visually salient stimulus properties. Default toolbox settings were used to assess visual salience and compute saliency maps. In order to establish the visual salience of faces within each stimulus, output saliency maps were Z-normalised and the average saliency value of the face AOI region was calculated. The face did not constitute the most salient aspect of any scene and average values were very low (black faces: *M* = 0.108, *SD* = 0.384; white faces: *M* = 0.093, *SD* = 0.418) relative to maximum Z-values, which were approximately 7 SDs for each image. An independent *t* test revealed no significant saliency differences between FaceRace stimulus sets (*t*(14) = 0.081, *p* = 0.936). 

## 4. Discussion

Infants did not show significant differences in attentional capture to own- and other-race faces, but once fixated, other-race faces held infants’ visual attention for longer. Investigating *detection*, we found that face detection saccadic latencies did not differ for own- and other-race faces, but a marginally higher number of own-race faces were detected. We also find a clear developmental progression in face orienting from six to twelve months, with older infants orienting to faces faster (attention *capture*) and fixating them for longer (attention *holding*). Older infants also showed improved face detection relative to younger infants, though all age groups detected faces in naturalistic scenes well above chance level, mirroring recent findings [19].

According to the prevailing account of face processing, faces are detected via a “quick and dirty” subcortical system (*Conspec*) that specialises in the rapid processing of low spatial frequencies such that preferential orienting occurs even for face-like configural stimuli [9,10]. Our findings are consistent with this model given that the race of the face stimuli did not impact the latency of infants’ face orienting. Whilst there is evidence the subcortical system may be sensitive to certain stimulus aspects such as contrast polarity [6], or the presence of biologically-relevant signals such as fearful expressions [42], our work suggests that faces in naturalistic scenes are rapidly oriented irrespective of their race, despite infants’ asymmetrical experience of race within a region [43].

The two-process theory also predicts that once a face is fixated, further processing occurs via a parallel cortical system (*Conlern*). It suggests that through experience to faces in the environment, cortical circuitry is specialised for higher-order processing. Concordantly, newborn infants are insensitive to race ([27, 33]; though see [10]), but begin to demonstrate differential attention to [21,37,38] and processing for [24,33] race as a specialisation for faces that occur more frequently within their immediate environment is acquired. Our findings are consistent with this account as, once fixated, other-race faces in scenes held infants’ attention for longer. We speculate that this asymmetry in attentional holding might be driven by an acquired cortical specialisation (*Conlern*) for own-race faces.

Previous work suggests that attentional preferences for race transition from an own-race familiarity preference at three months through a null preference at six months to another-race novelty preference at nine months [29,37]. This study’s findings suggest that an attentional bias toward other-race faces may emerge earlier (at six months), when face stimuli are embedded within a naturalistic environment. This indicates that context may play a role in the processing of the race of a face. Previous work has found enhanced detection when faces are embedded within naturalistic scenes compared to in grids or arrays [19]. While the rate of face detection was well above chance in this study, the proportions of detected faces were lower than for Kelly and colleagues [19] and the saccadic latencies were slower. In contrast to Kelly and colleagues [19] who presented the whole body in natural locations, the face stimuli used here were disembodied and situated in unexpected locations. This difference might account for the poorer face detection performance in our cohort, and further supports the role of the environmental context in guiding face detection. As noted in the introduction, the presentation of to-be-detected faces in isolation differs from the viewing of faces in the real world, where a body is also present. Yet this is a limitation that is also present in previous work using visual arrays and is an important strategy in the context of the current study, given the emphasis on the detection of faces, as opposed to people, per se. However, it would be insightful for future studies to explore the role of the body in guiding infants’ visual attention to own- and other-race faces.

Although we found infants were matched on orienting speed, we did find a small but significant effect of stimulus race on the number of faces detected, with a greater number of first-looks toward own-race (white) faces, which could indicate an own-race familiarity bias for face detection, especially as our saliency analysis suggested that differences in detection were not driven by low-level properties such as assessed by visual saliency analysis in this study. However, as noted in the introduction, this is the first study to investigate infants’ abilities to detect faces of different races within complex, real-world scenes and as such, future studies might explore the role of visual saliency and context more fully in own- and other-race face orienting.

## 5. Conclusions

In conclusion, the current findings demonstrate that six-, nine-, and twelve-month-old infants rapidly orient towards both own- and other-race faces, but once fixated, other-race faces hold their attention for longer. Collectively, these results are consistent with the predictions of the two-process model of face processing [9]. and with past work showing that a bias for faces strengthens across ontogeny see [11]. 

## Figures and Tables

**Table 1:** Definitions of variables.

***Attention Orienting***	***Detection***	‘First look’ i.e., the percentage of trials in which the infant’s first saccade is directed to a face AOI.
	***Capture***	‘Orienting speed’ i.e., the mean time taken to orient toward a face AOI following stimulus onset—not necessarily first fixation
***Attention Holding***		‘Face fixation duration’ i.e., the mean percentage of total stimulus looking time spent on a face AOI once it has been fixated

**Table 2:** Age (in days) and gender details of infant participants.

6 months (*n* = 50)	9 months (*n* = 73)	12 months (*n* = 49)
*M* = 184.5 range = 178–193 female = 23	*M* = 275.1 range = 268–282 female = 37	*M* = 368.2 range = 361–374 female = 29

## Supplementary Materials

The following are available online at https://www.mdpi.com/2076-3425/10/1/53/s1; Table S1: Face distance, finding and detection values for each experimental stimulus. Table S2: Face Fixation Onset Time (Attention Capture) and Post-First-Face Fixation Dwell Time (Attention Holding) results summary (standard deviations in parenthesis), Table S3: Number of faces detected and Face Detection Saccadic Latency results summary (standard deviations in parenthesis).

## References

[1] Haxby J.V., Hoffman E.A., Gobbini M.I. (2000). The distributed human neural system for face perception [Record Supplied By Publisher]. Trends Cogn. Sci..

[2] Crouzet S.M., Kirchner H., Thorpe S.J. (2010). Fast saccades toward faces: Face detection in just 100 ms. J. Vis..

[3] Johnson M.H., Dziurawiec S., Ellis H., Morton J. (1991). Newborns’ preferential tracking of face-like stimuli and its subsequent decline. Cognition.

[4] Turati C., Valenza E., Leo I., Simion F. (2005). Three-month-olds’ visual preference for faces and its underlying visual processing mechanisms. J. Exp. Child Psychol..

[5] Farroni T., Johnson M.H., Menon E., Zulian L., Faraguna D., Csibra G. (2005). Newborns’ preference for face-relevant stimuli: Effects of contrast polarity. Proc. Natl. Acad. Sci. USA.

[6] Valenza E., Simion F., Cassia V.M., Umiltà C. (1996). Face Preference at Birth. J. Exp. Psychol. Hum. Percept. Perform..

[7] Reid V.M., Dunn K., Young R.J., Amu J., Donovan T., Reissland N. (2017). The Human Fetus Preferentially Engages with Face-like Visual Stimuli. Curr. Biol..

[8] Scheel A.M., Ritchie S.J., Brown N.J.L., Jacques S.L. (2018). Methodological problems in a study of fetal visual perception. Curr. Biol..

[9] Johnson M.H., Senju A., Tomalski P. (2015). The two-process theory of face processing: Modifications based on two decades of data from infants and adults. Neurosci. Biobehav. Rev..

[10] Johnson M.H. (2005). Subcortical face processing. Nat. Rev. Neurosci..

[11] Leppänen J.M. (2016). Using Eye Tracking to Understand Infants’ Attentional Bias for Faces. Child Dev. Perspect..

[12] Elsabbagh M., Gliga T., Pickles A., Hudry K., Charman T., Johnson M.H. (2013). The development of face orienting mechanisms in infants at-risk for autism. Behav. Brain Res..

[13] Gliga T., Elsabbagh M., Andravizou A., Johnson M.H. (2009). Faces attract infants’ attention in complex displays. Infancy.

[14] Gluckman M., Johnson S.P. (2013). Attentional capture by social stimuli in young infants. Front. Psychol..

[15] Di Giorgio E., Turati C., Altoè G., Simion F. (2012). Face detection in complex visual displays: An eye-tracking study with 3- and 6-month-old infants and adults. J. Exp. Child Psychol..

[16] Jakobsen K.V., Umstead L., Simpson E.A. (2016). Efficient human face detection in infancy. Dev. Psychobiol..

[17] Amso D., Haas S., Markant J. (2014). An eye tracking investigation of developmental change in bottom-up attention orienting to faces in cluttered natural scenes. PLoS ONE.

[18] Frank M.C., Amso D., Johnson S.P. (2014). Visual search and attention to faces during early infancy. J. Exp. Child Psychol..

[19] Kelly D.J., Duarte S., Meary D., Bindemann M., Pascalis O. (2019). Infants rapidly detect human faces in complex naturalistic visual scenes. Dev. Sci..

[20] Quinn P.C., Lee K., Pascalis O. (2019). Face Processing in Infancy and Beyond: The Case of Social Categories. Annu. Rev. Psychol..

[21] Kelly D.J., Quinn P.C., Slater A.M., Lee K., Gibson A., Smith M., Ge L., Pascalis O. (2005). Three-month-olds, but not newborns, prefer own-race faces. Dev. Sci..

[22] Pascalis O., Kelly D.J. (2009). The origins of face processing in humans: Phylogeny and ontogeny. Perspect. Psychol. Sci..

[23] Bar-Haim Y., Ziv T., Lamy D., Hodes R.M. (2006). Nature and Nurture in Own-Race Face Processing. Psychol. Sci..

[24] Kelly D.J., Liu S., Ge L., Quinn P.C., Slater A.M., Lee K., Liu Q., Pascalis O. (2007). Cross-race preferences for same-race faces extend beyond the African versus Caucasian contrast in 3-month-old infants. Infancy.

[25] Anzures G., Quinn P.C., Pascalis O., Slater A.M., Lee K. (2010). Categorization, categorical perception, and asymmetry in infants’ representation of face race. Dev. Sci..

[26] Bindemann M., Burton A.M. (2009). The role of color in human face detection. Cogn. Sci..

[27] Bindemann M., Scheepers C., Ferguson H.J., Burton A.M. (2010). Face, Body, and Center of Gravity Mediate Person Detection in Natural Scenes. J. Exp. Psychol. Hum. Percept. Perform..

[28] Birmingham E., Bischof W.F., Kingstone A. (2009). Get real! Resolving the debate about equivalent social stimuli. Vis. Cogn..

[29] Fassbender I., Teubert M., Lohaus A. (2016). The development of preferences for own-race versus other-race faces in 3-, 6-and 9-month-old Caucasian infants. Eur. J. Dev. Psychol..

[30] Fysh M.C. (2018). Individual differences in the detection, matching and memory of faces. Cogn. Res. Princ. Implic..

[31] Hayden A., Bhatt R.S., Zieber N., Kangas A. (2009). Race-based perceptual asymmetries underlying face processing in infancy. Psychon. Bull. Rev..

[32] Holmqvist K., Nyström M., Mulvey F. (2012). Eye tracker data quality: What it is and how to measure it. Proc. Symp. Eye Track. Res. Appl..

[33] Kelly D.J., Liu S., Lee K., Quinn P.C., Pascalis O., Slater A.M., Ge L. (2009). Development of the other-race effect during infancy: Evidence toward universality?. J. Exp. Child Psychol..

[34] Kelly D.J., Quinn P.C., Slater A.M., Lee K., Ge L., Pascalis O. (2007). The other-race effect develops during infancy: Evidence of perceptual narrowing. Psychol. Sci..

[35] Sugden N.A., Marquis A.R. (2017). Meta-Analytic Review of the Development of Face Discrimination in Infancy: Face Race, Face Gender, Infant Age, and Methodology Moderate Face Discrimination. Psychol. Bull..

[36] Quinn P.C., Lee K., Pascalis O., Tanaka J.W. (2016). Narrowing in categorical responding to other-race face classes by infants. Dev. Sci..

[37] Liu S., Xiao W.S., Xiao N.G., Quinn P.C., Zhang Y., Chen H., Ge L., Pascalis O., Lee K. (2015). Development of visual preference for own-versus other-race faces in infancy. Dev. Psychol..

[38] Singarajah A., Chanley J., Gutierrez Y., Cordon Y., Nguyen B., Burakowski L., Johnson S.P. (2017). Infant attention to same- and other-race faces. Cognition.

[39] Levin D.T. (1996). Classifying faces by race: The structure of face categories. J. Exp. Psychol. Learn. Mem. Cogn..

[40] Minear M., Park D.C. (2004). A lifespan database of adult facial stimuli. Behav. Res. Methods Instrum. Comput..

[41] Walther D., Koch C. (2006). Modeling attention to salient proto-objects. Neural Netw..

[42] Peltola M.J., Hietanen J.K., Forssman L., Leppänen J.M. (2013). The emergence and stability of the attentional bias to fearful faces in infancy. Infancy.

[43] Office for National Statistics (2011). 2011 Census: Cultural Diversity in Kent.

