# Supplementary material for: Caucasian Infants’ Attentional Orienting to Own- and Other-Race Faces
Source: Brain Sci. 2020 Jan 17;10(1):53. doi: 10.3390/brainsci10010053 (PMC7016870; doi:10.3390/brainsci10010053)
Supplement: Supplementary file 1 [file brainsci-10-00053-s001.pdf]

Supplementary Information

Caucasian infants' attentional orienting to own- and other-race faces

Table S1. Face distance, finding and detection values for each experimental stimulus

Stimulus Index	Face Race	Distance (degrees of visual angle)	Distance Code	Number of Faces Detected	Number of Faces Found	Average Speed of Face Orienting (seconds)
1	Black	11.63	1	69	144	0.94
2	Black	13.44	2	59	115	1.54
3	Black	15.68	2	35	141	1.18
4	Black	16.55	3	41	143	1.15
5	Black	11.40	1	64	152	1.01
6	Black	12.79	1	51	148	0.61
7	Black	14.21	2	60	124	1.34
8	Black	15.34	2	51	143	1.22
9	White	13.91	2	65	149	0.83
10	White	17.06	3	47	136	0.80
11	White	12.80	1	53	95	1.36
12	White	10.92	1	50	94	1.78
13	White	12.95	1	76	146	0.85
14	White	11.84	1	81	147	1.05
15	White	17.11	3	51	148	1.05
16	White	14.75	2	53	136	1.09

Table S2. Face Fixation Onset Time (*Attention Capture*) and Post-First-Face Fixation Dwell Time (*Attention Holding*) results summary (standard deviations in parenthesis)

Age Group	Attention Capture (secs)		Attention Holding (%)	
	Black	White	Black	White
Six Months	1.24 (.61)	1.30 (.63)	53.87 (2.50)	42.27 (2.33)
Nine Months	1.13 (.44)	1.07 (.37)	58.84 (2.07)	51.96 (1.93)
Twelve Months	1.02 (.41)	.96 (.29)	59.38 (2.53)	54.24 (2.36)

Table S3. Number of faces detected and Face Detection Saccadic Latency results summary (standard deviations in parenthesis)

Age Group	Number of Faces Detected (max = 8 per race condition)		Face Detection Saccadic Latency (secs)	
	Black	White	Black	White
Six Months	2.04 (1.60)	2.54 (1.43)	.51 (.13)	.57 (.21)
Nine Months	2.52 (1.56)	2.64 (1.29)	.53 (.16)	.52 (.16)
Twelve Months	2.93 (1.72)	3.18 (1.70)	.52 (.16)	.51 (.17)
